# Exploring the formal assessment discussions in clinical nursing education: An observational study

**DOI:** 10.1186/s12912-022-00934-x

**Published:** 2022-06-16

**Authors:** Ingunn Aase, Kristin Akerjordet, Patrick Crookes, Christina T. Frøiland, Kristin A. Laugaland

**Affiliations:** 1 SHARE- Centre for Resilience in Healthcare, Faculty of Health Sciences, University of Stavanger, Kjell Arholms gate 41, N-4036 Stavanger, Norway; 2 School of Psychology, Faculty of the Arts, Social Sciences & Humanities, University of Wollongong, Wollongong, NSW Australia; 3 School of Nursing, Midwifery and Public health, University of Canberra, Canberra, Australia

**Keywords:** Clinical assessment discussions, Clinical education, Clinical placements in nursing homes, First-year nursing student, Observational study

## Abstract

**Introduction:**

According to EU standards, 50% of the bachelor education program in nursing should take place in clinical learning environments. Consequently, this calls for high quality supervision, where appropriate assessment strategies are vital to optimize students’ learning, growth, and professional development. Despite this, little is known about the formal assessment discussions taking place in clinical nursing education.

**Objective:**

The aim of this study was to explore the characteristics of the formal assessment discussions taking place during first-year students’ clinical education in nursing homes.

**Method:**

An exploratory qualitative study was performed. The data consist of passive participant observations of 24 assessment discussions (12 mid-term and 12 final assessments) with first-year nursing students (*n*=12), their assigned registered nurse mentors (*n*=12) and nurse educators (*n*=5). The study was conducted in three public nursing homes in a single Norwegian municipality. Data were subjected to thematic analysis. The findings were reported using the Standards for Reporting of Qualitative Research.

**Results:**

Three themes were identified regarding the characteristics of the formal assessment discussions: (1) adverse variability in structuring, weighting of theoretical content and pedagogical approach; (2) limited three-part dialogue constrains feedback and reflection; and (3) restricted grounds for assessment leave the nurse educators with a dominant role.

**Conclusion:**

These characteristic signal key areas of attention to improve formal assessment discussions to capitalize on unexploited learning opportunities.

## Background

This study focuses on formal assessment practice of nursing students in clinical education in nursing homes. Enabling nursing students to acquire professional competence through clinical education in a variety of healthcare settings is a cornerstone of contemporary nurse education programs [1]. According to EU standards, 50% of the bachelor education program should take place in clinical learning environments. Consequently, this calls for high-quality clinical supervision that includes appropriate assessment strategies, which are critical to optimize students’ learning, professional development, and personal growth [2].

Formal assessment of nursing students in clinical education serves two purposes: 1) to facilitate learning by enabling students to judge their own achievements more accurately and encourage their continuous learning process; and 2) to provide certification of achievements [3]. Accordingly, there are two approaches to assessment: formative assessment and summative assessment. *Formative assessment* is focused on the learning needs of each student, identifying areas in need of development, and providing feedback. Feedback is the central component of effective formative assessment. A *summative assessment* is a summary of a student’s achievements and judgement as to whether he/she has met the required learning outcomes [4, 5]. Student clinical placements are often assessed on a pass-fail basis, not by a letter grade [6].

The predominant clinical education model applied in nursing homes involves students being mentored and assessed by an RN and followed up by a nurse teacher [7]. The formal assessment during clinical education involves a partnership model where nursing students, their assigned Registered Nurse (RN) mentors and nurse educators, cooperate and share responsibility for facilitating and confirming the students’ achievement of expected learning outcomes [8]. However, substantial variations in assessment practices internationally and nationally have been reported, suggesting that the assessment practices of nursing students in clinical education lack consistency [2, 3, 9, 10]. Consequently, a variety of tools for assessing student’s clinical competence exist, and these tools depend on different definitions of clinical competence and the components to be assessed such as knowledge, technical care skills, attitudes, behaviours, clinical judgment, and critical thinking [11–13]. Several international researchers have argued that reliable and comparable assessment of nursing students’ knowledge and skill would benefit greatly from the development and consistent use of national competency assessment tools [2, 8, 14, 15]. In their discussion paper, Gjevjon et al. [16] highlighted the importance of assessing the students’ progression in clinical skills and ensuring that their nursing competence is in line with official requirements and professional expectations. This implies that student assessments cannot be limited to the period of a single clinical placement period.

Stakeholders have reported challenges with the assessment of students’ competence in clinical nursing education [2, 17–19]. RN mentors report that they have trouble providing feedback and assessing student competence because of the absence of clear guidelines and assessment criteria [17, 20]. RN mentors also experience having a passive and peripheral role during formal assessment discussions [18, 21]. Conversely, nursing students report feeling insecure due to power disparities in the assessment discussions; students perceive their RN mentors as much more powerful than they are [22]. Moreover, students report that the personal chemistry between them and their assigned RN mentor could lead to differential treatment [22]. In comparison, nurse educators report that it is challenging to make the students’ competence and learning processes visible, and to ensure fair and equitable assessment of students in clinical placement/education (e.g., [23]). Difficulties in understanding the concepts used to describe the student learning outcomes, the language used in the assessment tools, limited mentor competence in assessment and restricted academic-clinical collaboration have also been reported in the literature (e.g., [2, 24–26]). A systematic review of assessment of student competence found that the use of a valid and reliable assessment tool, with clear criteria and continued education and support for mentors is critical to quality in learning opportunities [3].

Formative assessment with feedback as key aspect is arguably one of the most important factors in terms of students’ learning, personal growth, and professional development in discussions of clinical assessment [24, 27]. A multilevel descriptive study [20] concluded that students often do not receive sufficient constructive feedback in or during formal assessment discussions. This is of major concern since clinical learning is considered a signature pedagogy in preparation of nursing students for real-world practice (e.g., [2, 28]). For workplace learning to be optimized, nursing students need to explore the complexity of the patients experience and to be able to discuss and evaluate patient care with others inside the clinical environment and in an academic setting (e.g., [29, 30]). The focus of assessment is to aid nursing students’ continuous learning process which requires constructive feedbacks and opportunities for reflections between nursing student, RN mentor, and nurse educator [3]. Formal assessment offers a potential opportunity to optimize nursing students’ learning outcomes by extending and transforming their professional knowledge dialectically. The explicit focus of assessment is therefore very important as students tend to concentrate on achieving the required competencies which they are aware will be assessed [2].

Internationally, emerging evidence shows that summative assessment of nursing students’ competence is a matter of concern across countries and educational institutions as previously stressed due to a) lack of consistency in use of methods and tool, b) its openness to subject bias, and c) the fact that the quality of assessment varies greatly [2, 3]. According to Helminen et al. [2], there are few studies of summative assessment, and as far as we know, no studies have explored the characteristics of these formal assessment discussions by observing what is really going on in this three-part dialogue between nursing students, RN mentors and nurse educators. There is therefore a need to further increase our knowledge and understanding of assessment discussions’ characteristics and how these discussions can enhance students’ learning (e.g., [2, 20, 21]). To fill this knowledge gap, the aim of this study was to explore the characteristics of the formal assessment discussions that take place during first-year students’ clinical education in a nursing home using observation. This is considered a novel methodological approach to explore this field in nursing education.

## Methods

### Design

The study applied a qualitative, exploratory design using passive observation [31] to explore the characteristics of the mid-term and final assessment discussions. Such observations allow the researcher to be present and identifiable, but the researcher does not participate or interact with the people being observed [31]. Observational research is, as previously stressed, a novel way to study assessment discussions as most studies have applied interview methods retrospectively, as a source for collecting data about assessment discussions [3, 20]. Observational research offers a rich, in-depth approach which in contrast to interviews allow the researcher to identify context-specific issues of importance, to learn what is taken for granted in a situation and to discover what is happening by watching and listening to arrive at new knowledge [31]. The observations of the formal assessment discussions were distributed among three of the researchers (IA, CF, KL) who individually carried out passive observations using a structured guide to ensure rigor [32]. The Standards for Reporting Qualitative Research (SPQR) were used.

### The context of the observed assessment practices

In this study, nursing home placements are part of eight weeks of mandatory clinical education during nursing students’ first academic year. In the observational study, a preceptorship model was applied in which students are mentored by a RN and followed up by a nurse educator [7]. In Norway, mentorship is an integral part of an RN’s work. This implies that the mentor does not receive financial compensation or require formal training in mentorship (e.g., at master level). The RN mentors are employed by the nursing homes and the nurse educators included are employed by the university. The nurse educators are responsible for coordinating the students’ learning, organizing the assessment discussions assessing the nursing students in clinical placement.

The clinical education system for the students in this study is comprised of two formal assessment discussions: the midterm discussion, with a summative assessment and a formative assessment, and the final assessment discussion, where the whole period is encapsulated up in a summative assessment [20]. The mid-term and final summative assessments take the form of a three-part dialogue among the nursing student, the RN mentor, and the nurse educator at the placement site. Prior to the assessment discussions the nursing student must write an evaluation of his or her learning. This written self-assessment must be sent to the nurse educator and RN mentor two days before the assessment discussion. There is no requirement for written preparation or documentation from the RN mentors. The university would assign the student a pass or fail grade based on six competence areas (i.e., professional ethics and legal-, cooperation-, and patient-centered nursing, pedagogical, management and learning competence) with the accompanying learning outcomes. All six competence areas and accompanying learning outcomes with a particular focus on fundamentals of care were well known by the researchers (IA, CF, KL) serving as important pre-understanding for data-collection and analysis. Beyond this, all the researchers had experience as nurse educators from the nursing home context and one of the researchers (CF) holds a Master of Science degree in gerontology.

### Setting and sample

The study was conducted in three public nursing homes within the same municipality in Western Norway as part of a larger research project: “Aiming for quality in nursing home care: Rethinking clinical supervision and assessment of nursing students in clinical studies” [19]. The nursing homes varied in patient numbers and staffing but were highly motivated to participate in the research project anchored in the top management team. Recruitment was based on a purposive, criterion-based sampling strategy [33] targeting the nursing students, RN mentors and nurse educators involved in assessment discussions. To make sure that the sample had the virtue of knowledge and expertise, RN with mentorship experiences from nursing homes were included ensuring diversity related to gender, age, and ethnicity. The nursing students and the nurse educators were recruited from the same university representing a distribution in age, gender, healthcare experience, and academic experience (See Table 1).

**Table 1:** Characteristics of participants

Participant groups	Age	Previous experience	Gender	Not Norwegian as mother language
Nursing students (*n*=12)	19 - 29 years	0- 2 years as healthcare assistants	12 females	1 of 12
RN mentors (*n*=12)	25- 53 years	3-23 year of experience as RN nurse	11 females, 1male	4 of 12
Nurse educators (*n*=5)	38- 66 years	3-33 years as educators	Females	1 of 5

Prior to data collection, approval was obtained from the university and from the nurse managers at the nursing homes enrolled in the study. In addition, an information meeting was held by the first and last author of this study, with the eligible nursing students during their pre-placement orientation week on campus, the RN mentors at the selected nursing home sites, and with the nurse educators responsible for overseeing nursing students on placement.

Invitations to participate in the study were then sent to eligible participants at the three public nursing homes. Nursing students were recruited first, before their assigned RN mentors and nurse educators were emailed with an invitation to participate to ensure the quality of the study sample. Two co-researchers working in two of the three enrolled nursing homes assisted in the recruitment of the RN mentors. A number of 45 allocated nursing students had their clinical placement at these three included public nursing homes. Of the 45 potential nursing students invited to participate, 12 consented. Their assigned RN mentors (*n*=12) and nurse educators (*n*=5) also agreed to participate. A summary of participant group characteristics is displayed in Table 1.

Four nursing students and four RN mentors enrolled from each nursing home. As nurse educators are responsible for overseeing several students during placements, fewer nurse educators than students and RN mentors agreed to participated. All but one of the participants, a RN mentor, were women. Out of 29 participants, six (one nursing student, one nurse educator and four RN mentors) did not have Norwegian as their mother language. None of the RNs had prior formal supervision competence and their experience with mentoring students ranged from 1-7 years. Seven of the 12 nursing students had healthcare experience prior to their placement period. Three of the five nurse educators held a PhD and the other two were lecturers with a master’s degree. Two of the nurse educators were overseeing students on nursing home placement for the first and second time, while the other three had several years of experience with nursing student placements within nursing homes. None of the nurse educators had expertise in gerontological nursing.

### Data collection

To allow a first-hand experience of the formal assessment discussion passive observation were used as the main source of data collection. The observations were carried out separately by three researchers (IA, CF, KL), all of whom are experienced qualitative researchers with a background in nursing, nursing education and nursing research. The researchers were all familiar faces from lectures at the University and as previously emphasized, as nurse educators in the public nursing home settings. At the beginning of each assessment observation, time was taken to create bond of trust between the participants to reduce contextual stress. The observations were based on a structured observation guide (See Attachment 1). The observation guide contained relatively broad predefined categories: structure, content, duration, interaction, dialogue, and feedback. These predefined categories were used to guide and support the recording and notetaking process allowing space for spontaneous aspects of the formal assessment discussions [31]. The guide was based on the aim of the study and informed by the literature.

During the observations, each of the three researchers sat on chairs in a corner of the room (two-three meters away) to observe the interaction and listen unobtrusively to the assessment discussions during the study to reduce students’ experience of stress contextually. Observational notes were taken discreetly, according to the structured guide and combined descriptions and personal impressions [34]. Summaries, including reflective notes, were written in electronic format directly after the observations. The observations were conducted alongside the clinical placements in February and March 2019 with a duration of about 60 minutes on average. The choice of passive observation using three researchers was both of pragmatic and scientific reasons: a) to ensure that data was collected timely at colliding times of assessment meetings, and b) to verify the observed notes supported by triangulation during analysis and the interpretation process [33].

### Data Analysis

**Fig. 1:**
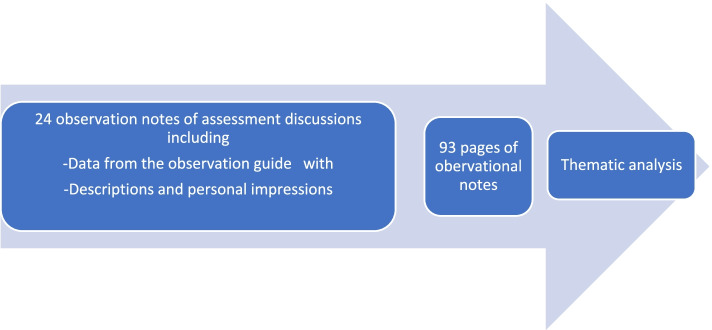
Analysis process

Braun and Clarke’s [35] approach to thematic analysis was used to analyze the observational notes and summary transcripts. The thematic analysis was guided by the aim of the study and followed the six steps described by Braun and Clarke [35]: (1) becoming familiar with the data; (2) generating initial codes; (3) searching for themes; (4) reviewing themes; (5) defining and naming themes; and (6) producing the report. The 93 pages of observational notes were read independently by three of the researchers (IA, CF, KL) to obtain an overall impression of the dataset (See Fig. 1: Analysis process).

All the observational notes, both from mid-term- and final assessment discussions, were then compared and analyzed as a single dataset with attention to similarities and differences by generating initial codes and searching for themes. The reason for merging the data set was that the mid-term and final assessment discussions overlapped in terms of initial codes and emerging themes contributing to a deeper understanding of the results. The researchers IA, CF and KL met several times to discuss the coding process to finalize the preliminary themes. All authors revised, defined, and identified three key themes reaching consensus. This means that all authors contributed to analytic integrity by rich discussions throughout the process of analysis, clarification, validation, and dissemination of results [36]. The study also provides a description of the context of the formal assessment discussions, participants enrolled, data collection and the analysis process allowing the readers to evaluate the authenticity and transferability of the research evidence [36].

### Ethical considerations

The study was submitted to The *Regional Committees Research Ethics in Norway who found that the study was not regulated by the Health Research Act, since no health or patient data is registered. The study adhered to general ethical principles laid down by The National Committee for Medical & Health Research Ethics in Norway. In addition, the principles of confidentiality, voluntary participation and informed consent were applied by following the World Medical Association’ s Declaration of Helsinki. All participants gave their written informed consent they* were informed about the right to withdraw from the study at any point. The nursing students were made aware that participation or non-participation would not affect other aspects of their clinical placement/education period or give them any advantages or disadvantages on their educational path. *The study was approved by The Norwegian Centre for Research Data in two phases (Phase 1: NSD, ID 61309; Phase 2: NSD, ID 489776).*

All methods were performed in accordance with relevant guidelines and regulations.

## Results

The analysis identified three themes that describe the characteristics of the formal assessment discussions taking place during first-year nursing students’ clinical education in nursing homes: (1) Adverse variability in structuring, weighting of theoretical content, and pedagogical approach, (2) Limited three-part dialogue constrains feedback and reflection, and (3) Restricted grounds for assessment leave the nurse educators with a dominant role. These themes are now presented.

### Theme 1: Adverse variability in structuring, weighting of theoretical content and pedagogical approach

This theme illuminates adverse variability in the nurse educators structuring of the discussion, weighting of theoretical content (e.g., bridging of theory and practice), and the pedagogical approach applied across the assessment discussions. Some nurse educators went through each competence area and the accompanying learning outcomes, strictly following the sequence adopted in the assessment form. Others adopted a more flexible sequence of progression guided by the discussion. The latter approach led to frequent shifts in focus between competence areas; the observation notes described that students and RN mentors found it difficult to follow the discussion and the assessment process. For example, the observational notes illuminated that a student commented that she felt insecure because the nurse educator appeared to write notes pertaining to one competence area while talking about another one.

The data exposed variations in the nurse educators’ emphases and weighting of bridging theory and practice. While some nurse educators asked theoretical questions to gauge the students’ knowledge and to help students to link theory and practice, others nurse educators did not. An absence of theoretical questions was observed in several assessment discussions. Additionally, the nurse educators’ knowledge and referral to the course curriculum varied. Some nurse educators seemed to be familiar with the course curriculum and mentioned the theoretical subjects taught to the students’ pre-placement, other nurse educators refrained from discussing theoretical issues. The weighting of geriatric nursing was limited in the assessment discussions.

The nurse educators varied in their pedagogical approach across the assessment discussions. Some nurse educators asked open questions inviting the students to self-evaluate and reflect on their own performance and development before approaching and inviting the RN mentor to provide input. Other nurse educators adopted a more confirmative approach, asking closed-ended questions, and reading aloud from the student’s written self-assessment before asking the RN mentors to just confirm the nurse educators’ impressions and evaluations with “yes” or “no” answers.

### Theme 2: Limited three-part dialogue constrains feedback and reflection

The second category illuminates limited participation of all three parties in the formal assessment discussions. This was observed as constraining feedback and reflection. Several possible impediments to the dialogue were language barriers, interruptions, preparedness of students and RN mentors for the discussions, justification for assessing the students, and the students’ reported level of stress. There were variations in the way both nurse educators and RN mentors conveyed their feedback to the students. Several of the RN mentors were observed to have assumed a passive, peripheral role, sitting in the sidelines and saying very little. When addressed by the nurse educator, the RN mentors tended to respond with yes/no answers.

Language barriers related to understanding of the learning outcomes to be assessed appeared to hamper the dialogue. On several occasions the RN mentors who did not have Norwegian as their mother language, expressed that it was difficult to fully understand the language used in the assessment form, so they did not know what was required during the assessment. We therefore observed that the nurse educators often took time to explain and “translate” the concepts used to describe the student learning outcomes to ensure mutual understanding.

Interruptions during the assessment discussions interfered with the dialogue. In several of the assessment discussions the RN mentors took phone calls that required them to leave the assessment discussions, sometimes for extended periods of time. This meant that the nurse educator later had to update the RN mentor on what had been covered in her/his absence.

Preparedness of students and RN mentors for the assessment discussion varied. Some students brought their self-assessment document with them to the meeting, others came empty-handed. Some but not all RN mentors brought a hard copy of the student’s self-assessment document. Some RN mentors admitted that they had been too busy to read the self-assessment before the meeting.

Based on body language interpretation, several students appeared nervous during the assessment discussions. The observational notes showed that some of the students later in this assessment discussion confirmed their self-perceived stress by expressing during the assessment discussions that they had had dreams or nightmares about these assessment discussions. The observational notes also illuminated that some students expressed during the assessment discussion that they did not know what would be brought up in these discussions and that they were afraid of failing their clinical placement. The three-part dialogue to a great extent focused on the student competence achievements to pass or fail the clinical placement and less attention providing the student with ability to reflect for enhancing learning.

### Theme 3: Restricted grounds for assessment leaves the nurse educators with a dominating role

Limited dialogue and engagement from students and RN mentors often left the nurse educators with restricted basis for assessment and thus gave them a dominant role in the assessment discussions. RN mentors seemed to have insufficient information to assess their students’ performance, learning and development stressing that they had not spent significant amounts of time observing them during the placement period. On several occasions RN mentors expressed that due to sick leave, administrative tasks, and alternating shifts, they had spent only a handful of workdays with their students. The observational notes revealed that some RN mentors expressed that they had to base their formal assessments of student performance on these assessment discussions mainly. Several RN mentors expressed frustration with limited nurse coverage which could impede their mentorship and assessment practices and the amount of student follow-up during placement. Because of limited input from the RN mentors, the researchers observed that the nurse educators had to base their evaluations on students’ written self-assessments. Some nurse educators gave weight to the students’ capacity for self-evaluation. The quality, amount and content of the written self-assessment was observed to be influential for determining the students’ strengths and weaknesses and whether the students passed or failed their competence areas. Our observational notes showed that some students struggled to pass because their written self-assessments were not comprehensive enough. In the formal assessment document, there are fields for ‘strengths and areas of growth /improvement’. It was variations in how much was written beyond the mark for approved or not approved and pass or fall. This implies that some assessment discussions were marked by ticking a box rather than engaging in a reflective dialogue.

## Discussion

The findings of this exploratory observational study suggest that the formal assessment discussions for first-year nursing students are characterized by lack of conformity - referred to as *adverse variability-* regarding structure, theoretical content and the pedagogical approach. The limited *three-part dialogue* appeared to constrain feedback and critical reflections to enhance students clinical learning opportunities, leaving the nurse educators with a dominant role and a *restricted ground for assessment*. Increased awareness is therefore required to improve formal assessment discussions to capitalize on unexploited learning opportunities.

### Unexploited learning opportunities

Formal assessment discussions are expected to optimize nursing students’ clinical learning outcomes by extending and transforming their professional knowledge. Our findings illuminate adverse variability in nurse educators’ pedagogical approach and the weighting of theoretical content during formal assessment, which may reduce the students’ ability to learn. This is of major concern since the assessment discussion should be clear and systematic encouraging student’s continuous reflecting and learning process. Both nursing students and nurse mentors seem to need more knowledge of what a formal assessment discussion consists of to reduce unpredictability (e.g., familiarizing with the expected learning outcomes, the assessment criteria, and the context) and to optimize clinical learning. Regarding knowledge or referral to theoretical teaching prior to clinical placements, nurse educators may fail to provide consistency for first-year students during formal assessment; supporting them in bridging theory and practice which is essential to their learning and professional development. These findings resonate with an integrative review which indicated that orientation programs, mentor support, clear role expectations, and ongoing feedback on performance are essential for academic organizations to retain excellent nursing faculty [37]. This highlights the importance of nurse educators' awareness and active involvement in assessment discussions [38, 39] to provide academic support and guidance of students' theory-based assignments [40]. A critical question is whether the nurse mentors’ competence and active role have been acknowledged sufficiently in the formal assessment discussions? Particularly when our research revealed that gerontological questions were hardly reflected upon to support students clinical learning in nursing homes.

Critical reflections in the assessment discussions were also limited since some nurse educators in this study mostly did not ask for reflections from the students. This is of major concern since critical reflection is a pedagogical way to bridge theory with clinical experience and to tap into unexploited learning opportunities by strengthening the students’ reflection skills and knowledge in the assessment discussions [41]. Critical reflection in clinical setting and education is known to assist students in the acquisition of necessary skills and competencies [42].

Encouraging students to reflect on clinical learning experiences contextually and having clearer guidelines for formal assessment discussions in educational nursing programs may be both necessary and important for exploring unexploited learning opportunities. Our findings imply that education programs may increase students’ learning opportunity by decreasing the variability in structure, the weighting of theoretical content and the pedagogical approach applied in clinical education. Increased awareness of assessment by having a common understanding of how the assessment should be managed and what the assessment criteria are therefore considered of importance (e.g., [17]). Research is however needed to explore the relationship between nurse educators’ clinical expertise, competence, pedagogical skills set and students’ learning outcomes [26]. Our findings suggest that measures for preparing nurse educators for better theoretical knowledge and pedagogical approach require further development, and that this could be done with i.e., online educational support. Further research should therefore explore and extend our understanding of the need for improvement in structure, theoretical content, and pedagogical approach in formal assessment discussions.

### Hampered three-part dialogue

The study findings illuminate that a hampered three-part dialogue makes it hard to offer feedback and engage in reflection. This interfered with the amount of learning potential during formal assessment in clinical education. Many factors affect the degree of interaction in the three-part dialogue. For example, our findings imply that RN mentors gave little feedback to the students in the assessment discussions because they had not spent a significant amount of time with students. A consequence of inadequate feedback in the assessment discussions may limited the clinical learning potential for the nursing students. Formative assessment is arguably one of the most important and influential factors for students’ learning, personal growth, and professional development in their clinical assessment discussions [27]. According to our findings, formative process assessment is not always used properly, even though it is highly recommended in the literature (e.g., [3, 4]). Our study shows that enhanced focus on formative assessment in the nurse educational program and further research using different methodology is of significance to extend our knowledge of the formative process assessment related to clinical learning. Overall, our findings indicate that there are room for improvement in the way that RN mentors participate in the assessment discussions. RN mentors and nurse educators need to increase their knowledge in how giving constructive and substantive feedback and how to encourage students to reflect critically on their learning through the formal assessment discussions. The findings also suggest that linguistic challenges associated with an internationally diverse workforce among RN mentors may constrain assessments of student competence. These findings are consistent with other studies that concluded that understanding the language and meaning of the concepts used in the assessment document was difficult and might have resulted in a peripheral and passive role of the RN nurses [18, 21, 26]. These linguistic challenges and the marginalization of RN nurses may be other causes of the limited dialogue requiring a better pedagogical preparation to nursing students’ clinical placement in nursing homes.

The results from our study also illuminated that the students appeared nervous before and during the assessment discussions. This anxiety could make the discussions difficult and stressful for them. Other studies mentioned the need for more predictability to reduce their stress, so they feel more secure [22, 43]. Similar findings are described in the study of nursing students’ experiences with clinical placement in nursing homes, where Laugaland et al. [19] highlighted the vulnerability of being a first-year student. The students need to be informed about the purpose of the formal assessment discussions, so they prepare for them and feel less anxious consequently. This may give them a better basis for and openness to learning in the three-part dialogue. Enhanced focus on students’ stress in the assessment discussions and further research on the consequences of stress for learning in the assessment discussions are therefore required.

The hampered three-part dialogue, indicated in our results, often left the nurse educators with restricted basis for assessing the nursing students as well as having a dominate role in the discussions. The cooperation between the nurse educators and the RN mentors was also limited related to both interruptions in the assessment discussion and little input from the RN mentors which is not ideal or desirable for the formal assessment discussions to support students clinical learning, also considered as an unexploited learning opportunity. Wu et al. [10] describe clinical assessment as a robust activity which requires collaboration between clinical partners and academia to enhance the clinical experiences of students. Our research indicates a need for increased collaboration between educational programs and clinical placements in nursing homes. One way to increase the collaboration and giving the RN mentor a more active role, may be to give the RN mentors dedicated time and compensation for mentoring students including access to academic courses in clinical mentoring. Another way to increase this collaboration may be that the leaders in the nursing homes let the RN mentors prioritize the supervision of the students during the clinical placement period. Future research should extend and explore measures to strengthen the collaboration without giving the nurse educators too much of a say in assessment discussions.

### Methodology considerations

This study has a strength by using passive observation as method in exploring and describing the formal assessment discussion using a novel approach to expand and deepen our knowledge in this research field.

A methodological consideration may be that the researcher claimed to be a passive observer of an interaction, but an observer’s presence is itself a significant part of the interaction. Participants might be uncomfortable and stressed seeing a stranger silently sitting in the corner and taking notes [31]. On the other hand, the researchers were familiar faces to the nursing students acknowledged by some of them being comforted with the researchers’ presence feeling more secure contextually. A critical question however is whether use of video recordings would provide richer data. Video recordings allow for the capturing and recording of interactions in the assessment discussions as they occur naturally without many disturbances and because they allow for repeated viewing and detailed analysis [44]. Frequencies of questions and answers in the tree-part dialogue might also have been counted to for example confirm the observed dominating role of the nurse educator. This was discussed but decided that it was not practical or possible contextually.

Video recording with no researcher present might also reduce the participants’ stress, but on the other hand also perceived as more threatening.

The way of collecting and analyzing the data by using three researchers should also be reconsidered. The data was collected separately by three researchers to ensure that the data was collected timely without any abruption. A limitation may be that all three researchers had similar educational background and experiences from formal assessment discussions in nursing homes. Their preconceptions might have influenced the results. Yet, the researchers were not involved in nursing students’ clinical placement period. To control for research bias, the data analysis process applied triangulation; two of the authors who were not actively involved in the observations, reflected upon the results, providing a basis for checking interpretations to strengthen their trustworthiness [45].

## Conclusions and implications

Adverse variability in structuring and weighting of theoretical content and pedagogical approach, a hampered three-part dialogue and limited basis for assessment, all lead to unexploited learning opportunities. These characteristic signal key areas of attention to optimize the learning potential of these formal assessment discussions.

Higher education is in a unique position to lay the ground for improvements of formal assessment discussions. Educational nursing programs may increase students’ learning opportunity by using a structured guide, decreasing the variability in the weighting of theoretical content and the pedagogical approach applied in clinical placement in nursing homes. The nursing educational program should therefore find ways to increase the collaboration between nurse educators, nursing students and RN mentors to improve feedback, critical reflections, and clinical learning, thereby limiting the nurse educators dominating role in the formal assessment discussions in nursing homes.

## Data Availability

Original de-identified data of the study will be stored at the Norwegian Centre of Research Data subsequent to completion of the project. Original de- identified data can be requested from the corresponding author upon reasonable request.

## Abbreviation

RN: Registered nurse
